# Effect of Carrier Agents on the Physicochemical and Technofunctional Properties and Antioxidant Capacity of Freeze-Dried Pomegranate Juice (*Punica granatum*) Powder

**DOI:** 10.3390/foods9101388

**Published:** 2020-10-01

**Authors:** Adegoke Olusesan Adetoro, Umezuruike Linus Opara, Olaniyi Amos Fawole

**Affiliations:** 1 Postharvest Technology Research Laboratory, South African Research Chair in Postharvest Technology, Department of Horticultural Science, Stellenbosch University, Private Bag X1, Stellenbosch 7602, South Africa; 21412944@sun.ac.za (A.O.A.); Opara@sun.ac.za (U.L.O.); 2 Postharvest Research Laboratory, Department of Botany and Plant Biotechnology, University of Johannesburg, P.O. Box 524, Auckland Park, Johannesburg 2006, South Africa

**Keywords:** total soluble solids, particle size distribution, total anthocyanin content, antioxidant capacity

## Abstract

The physicochemical and technofunctional properties and antioxidant capacity of freeze-dried “Wonderful” pomegranate juice powder (PJP), produced with different carrier agents, were investigated. Powders were produced using maltodextrin, gum Arabic, and waxy starch as carrier agents and characterised by scanning electron microscopy (SEM) and particle size distribution. Results showed that PJP produced with maltodextrin had the highest yield (46.6%), followed by gum arabic (40.6%), while waxy starch had the least yield (35.4%). Powders produced with maltodextrin (96.5%) and gum arabic (96.1%) were highly soluble, which indicates better reconstitution properties. Waxy starch-added PJP had the lowest hygroscopicity (4.7%), which offers good stability during storage and a lower degree of caking compared to maltodextrin (10.2%) and gum arabic (12.6%) powders. Powders obtained from maltodextrin and gum arabic exhibited larger particle diameters ranging between 12 to 120 µm while the lowest particle diameter range was with powders formed from waxy starch (8–40 µm). Freeze-dried pomegranate powder produced with maltodextrin retained more redness (a*) by approximately 44%, compared to gum arabic. Similarly, PJP with maltodextrin and gum arabic had higher total soluble solids (10.3 and 10.4 °Brix), respectively. Total anthocyanin content was 54% more in PJP with maltodextrin than waxy starch PJP. Similarly, the powder produced with maltodextrin had higher radical scavenging activity (33.19 mM TE/g dry matter; DM) compared to gum arabic (28.45 mM TE/g DM) and waxy starch (26.96 mM TE/g DM). Overall, maltodextrin reflected the most suitable carrier agent to produce PJP.

## 1. Introduction

Pomegranate (*Punica granatum* L.) belongs to the Punicaceae family and is widely grown in many parts of the world, such as Europe, Asia, North Africa, the Mediterranean basin and, in recent times, South Africa [1,2]. The increased commercial production of pomegranate from 828 ha in 2016 to 1024 ha in 2019 is highly related to its rich phytochemical compounds in the edible part of the fruit [3,4]. Polyphenols, such as flavonoids, condensed tannins and hydrolysable tannins, are major components found in pomegranate arils or juice [5]. They are the major source of protective compounds that work against the damaging effects of free radicals [6]. Pomegranate fruit is mostly consumed as fresh juice, flavourings, colourings, concentrates and jellies for recipes [7]. Epidemiological studies have associated the consumption of pomegranate fruit to a reduced risk of coronary heart disease, diseases that are not transmissible, such as cancer, and diabetes as a result of its high antioxidant capacity [8,9]. Pomegranate fruit was noted to be actively used in folk medicine as a result of its high anthocyanin compositions of cyanidin, delphinidin and pelargonidin, which are attributed to the red colour of fruit and aril [10]. It is essentially useful in the cure of many parasitic diseases such as ulcers, diarrhoea, acidosis, dysentery, and haemorrhage [11].

Due to its health and nutritional benefits, pomegranate fruit is in demand throughout the year. Thus, the food industry desires a novel process aimed at increasing the shelf-life and improving the pigment stability of pomegranate products. The drying of fruit juice into powder form is a novel way to extend the shelf-life [12]. As opposed to dried pomegranate arils, pomegranate juice powders have the advantages of easier storage and distribution. Furthermore, the powders can be used as an ingredient to formulate foods. Spray drying is a commonly used method in many food industries for producing food powders and agglomerates [13,14]. Along with being an attractive feature of this technological process, the scarce heat damage to the product is essential [15]. Another challenging factor during spray drying is the clogging of nozzles, especially when drying sugar and acid-rich foods such as honey and natural fruit juices [16]. The low operating conditions involved in freeze-drying could be an appropriate drying method to produce niche fruit powders from sugar and acid-rich fruit like pomegranate. Freeze-drying is one of the techniques used to produce high-value powder products [12]. It is a method that results in high-quality dehydrated products due to the low operating temperatures required in the process and the absence of liquid water [17]. This method reduces thermal damage of nutrients and preserves flavour and colour components of the product [18].

Studies have reported some factors to be considered during the production of fruit powders: the stickiness of powder particles and safe handling and storage [19]. Stickiness during drying is mainly due to the high content of sugars such as fructose, glucose, sucrose and acid materials; for example, organic acids such as citric, malic and tartaric acids, which are attributed low molecular weight, contribute more than 90% of solids in fruit juices [20,21]. In order to overcome the sticky behaviour of fruit juice powder, high molecular weight carriers or drying aids such as maltodextrin, gum arabic, waxy starch, pectin, vegetable fibres, and starches as encapsulation agents are added [16,21,22,23]. Studies have shown that carrier agents further preserve some sensitive properties of the food material, such as carotenoids and flavours, and minimise volatile and reactive properties. For instance, mango juice powder obtained through maltodextrin, gum arabic, and waxy starch resulted in characteristic amorphous particles [24,25,26,27].

Yousefi et al. [28] reported that gum arabic showed a high colour change and increased glass transition temperature (Tg) of pomegranate powder. Similarly, Seerangurayar et al. [19] reported that carrier-agent-added date powders had lower hygroscopicity, which offers good storage stability. Fazaeli et al. [18] reported that additives enhanced the properties of the final product as a result of an increase in Tg and contributed to the high stability of quality attributes of black mulberry juice powder during storage. However, there are limited scientific studies specifically on the processing of pomegranate juice with the use of a freeze-dryer. To further examine the field of application for pomegranate products, this work investigates the freeze-drying of pomegranate juice to evaluate the influence of different carrier agents (maltodextrin, gum arabic and waxy starch) on the physicochemical and technofunctional properties and antioxidant activities of the powders.

## 2. Materials and Methods

### 2.1. Raw Material and Sample Preparation

Pomegranate fruit (cv. Wonderful) were harvested at commercial maturity from Blydeverwacht orchard, Wellington, South Africa. The fruit were sorted for uniformity of size, shape, and colour and transported in an air-conditioned vehicle to the Postharvest Technology Laboratory at Stellenbosch University. Fruits were washed, and the juice was extracted using a hand-operated domestic press and frozen at −20 °C for about 24 h.

The fresh juice was thawed and clarified using a centrifuge system (5810 R Eppendorf AG, Hamburg, Germany) at 10,000 rpm for 20 min. The cold, sterile single strength clarified juice with 16.2 °Brix (total soluble solids) was diluted and standardised with distilled water to 12 °Brix and rapidly frozen at −80 °C until experiments were carried out.

In order to obtain a flowable powder from pomegranate juice, a preliminary study was conducted to investigate the amount of carrier that would be added to the pomegranate juice. Each of the carriers (maltodextrin—Sigma Aldrich Co., St. Louis, MO, USA; gum arabic—Sigma Aldrich Co., France; waxy starch—Sigma Aldrich Co., USA) was incorporated in 100 mL pomegranate juice at a range between 10 to 40 g to select a suitable concentration of carrier agent. A 30 g concentration of (maltodextrin, gum arabic or waxy starch)/100 mL pomegranate juice was observed to produce a flowable powder, which was added after standardisation. The mixture was homogenised using a laboratory homogeniser for 5 min [28].

### 2.2. Freeze-Drying Procedure

**Figure 1:**
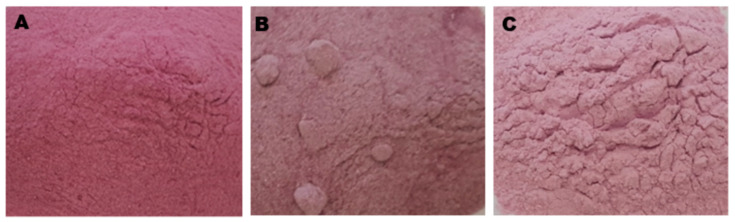
Freeze-dried pomegranate powder produced with (**A**) maltodextrin, (**B**) gum arabic and (**C**) waxy starch.

The pomegranate juice was placed in a 90-mL specimen jar and frozen in a static-air freezer at −80 °C. Freeze-drying of frozen samples was carried out in triplicates. A specimen jar containing the samples was carefully taken to a laboratory-scale freeze-dryer (VirTis Co., Gardiner, NY, USA) operating at condenser temperature −85 °C and pressure 18 mTorr and drying continued for 72 h. Dried samples were removed from the freeze-dryer and ground by electrical blender into free-flowing powder (Figure 1). The pomegranate juice powders (PJPs) were transferred and sealed in plastic bags in a desiccator that contained phosphorus pentoxide to prevent moisture absorption from the surrounding air until further analysis.

### 2.3. Yield, Water Activity and Physicochemical Attributes of PJP

#### 2.3.1. Powder Yield Determination

The percentage yield of powder was calculated based on the fresh weight [29]

(1) $$\mathrm{Yield}\left(\%\right)=\frac{\mathrm{Weight}\;\mathrm{of}\;\mathrm{powder}\;\left(g\right)}{\mathrm{Fresh}\;\mathrm{weight}\;\left(g\right)}\;\times \;100$$

#### 2.3.2. Determination of Water Activity and Moisture Content

The water activity (*a*_w_) of PJP was determined with an electronic dew point water activity meter (CH 8853 Novasina AG, Lachen, Switzerland). The final moisture content of the PJP was measured using a moisture analyser (KERN DBS 60-3 Balingen, Germany) at 120 °C.

#### 2.3.3. Colour Measurement

Colour of PJP was determined by direct reading using a chromometer (Minolta model CR-200, Osaka, Japan) to obtain the colour values: L* (brightness/darkness), a* (redness/greenness), and b* (yellowness/blueness). The measurements were taken at three different times from a colourless petri dish and averaged. The maximum for ‘L*’ value is 100 (white), and the minimum is zero (black). The colour attributes chroma C*, hue angle *h*° and total colour difference (TCD) were calculated [1,30].

(2) $$h^{o}=\tan^{-1}\frac{b^{*}}{a^{*}}$$

(3) $$C^{*}=\sqrt{a^{*2}+b^{*2}}$$

(4)  TCD=((ΔL*)2+(Δa*)2+(Δb*)2)12

L*, a* and b* represent the value after drying at each treatment level and results were expressed as means ± SE of the determinations obtained.

#### 2.3.4. Determination of Total Soluble Solids (TSSs), Titratable Acidity (TA) and pH

Five grams of PJP were extracted in 50 mL distilled water. For 5 min, the mixture was vortexed with the use of a vortex and sonicated for 15 min in an ultrasonic bath (Separation Scientific, Cape Town, South Africa). This was followed by centrifugation at 10,000 rpm for 25 min and recovery of the supernatant for TSS, TA and pH measurements. TSS measurement was determined with the use of a digital hand refractometer (model PT-32; ATAGO, Tokyo, Japan) blanked with distilled water. For TA, 2 mL of supernatant was diluted in 70 mL of distilled water and titrated against 0.2 N of sodium hydroxide (NaOH) to a pH of 8.2 with the use of a Metrohm 862 compact titrosampler (Herisau, Switzerland).

### 2.4. Technofunctional Characterisation of PJP

#### 2.4.1. Solubility

Solubility (%) was determined using the Eastman and Moore method [24] and modified slightly. One gram of the sample was uniformly dispersed in H_2_O of 50 mL and distilled in a vortex for 30 s. At 3000 rpm for 5 min under 25 °C, the solution was carefully placed in a tube and centrifuged. A 25 mL aliquot of the supernatant was transferred to preweighed Petri dishes and the sample was immediately dried at 105 °C for 5 h. Solubility (%) was determined by subtracting the initial weight from the final weight divided by the initial weight.

#### 2.4.2. Hygroscopicity

Hygroscopicity was calculated according to [31], with slight modifications. Two grams of the sample were placed inside a hermetic bottle that was controlled with NaCl-saturated solution in a constant relative humidity chamber (MLR-352 H Versatile Environmental Test Chamber, Kyoto, Japan) set at 68.9% RH and 25 °C [32]. The weight of the sample was calculated to validate the condition for equilibrium between the samples and the environment. The hygroscopicity was expressed as % moisture on wet basis (w.b.).

#### 2.4.3. Bulk Density

In a 100 mL graduated cylinder, PJP (20 g) was weighed and carefully dropped 10 times from a height of 15 cm using a rubber mat. The bulk density was determined by the division of the mass of powder and the volume estimated from the cylinder [33].

#### 2.4.4. Water- and Oil-Holding Capacity Determination

According to Jalal et al. [34], the water-holding capacity (WHC) and oil-holding capacity (OHC) of PJP were calculated. A mixture of 25 mL distilled water or sunflower oil and 250 mg of dry sample were slightly vortexed and left at room temperature for 1 h. The solution was placed in a tube and centrifuged at 4000 rpm for 10 min at 25 °C, after which the residue was weighed. The water/oil holding capacity was expressed as g of water/oil held per g of sample. The formula to calculate WHC/OHC is as follows:

(5) $$\mathrm{WHC}/\mathrm{OHC}\;(g/g)=\frac{\mathrm{residue}\;\mathrm{fresh}\;\mathrm{weight}-\mathrm{residue}\;\mathrm{dry}\;\mathrm{weight}}{\mathrm{residue}\;\mathrm{dry}\;\mathrm{weight}}$$

#### 2.4.5. Particle Size Distribution

The particle size of the powder was determined with the use of a laser light diffraction instrument (Mastersizer S, model MAM 5005; Malvern Instruments, Malvern, UK). Under magnetic agitation, a small amount of PJP was homogenised in 99% isopropanol, following careful monitoring of the distributed particle size, which was taken in three successive measurements. De Brouckere’s mean diameter was used to express the particle size and the mean diameter over the volume distribution. This is mostly used to characterise a particle [35].

#### 2.4.6. Microstructure

The microstructure of PJP was examined with the use of a scanning electron microscope (X-Max 51, Oxford Instruments, Concord, MA, USA). SEM images of powder were obtained from uniformly mixed powder samples. Under a high vacuum condition, the samples were coated with a very thin layer of gold. This is often used to provide a reflective surface for the electron beam. The gold coating was carried out in a sputter coater (ACE200 LEICA Mikrosysteme GmbH, Vienna, Austria) under a low vacuum condition while inert argon gas was present. Subsequent viewing of the gold-coated samples was carried out under the microscope.

### 2.5. Phenolic Contents and Antioxidant Capacity

#### 2.5.1. Determination of Total Phenolic Content (TPC)

TPC was determined by the Folin-Ciocalteu method using a methanolic extract of PJP [1]. In a test tube, the supernatant (0.05 mL) was mixed with 0.45 mL (50% methanol), followed by adding 0.5 mL Folin-Ciocalteu after 2 min. The mixture was then vortexed and kept in the dark for 10 min before adding 2% Na_2_CO_3_ and further incubation for 40 min in the dark. The absorbance of each sample was read at 520 nm in a UV-vis spectrophotometer (Thermo Scientific Technologies, Madison, WI, USA) against a blank containing 50% methanol. Absorbance was compared with a standard curve (Gallic acid, 0–10 mg), and results were expressed as mg gallic acid equivalent per gram dry matter (mg GAE/g DM).

#### 2.5.2. Total Anthocyanin Content

Total anthocyanin content (TAC) was quantified differentially by using the pH method [36]. In triplicates, 1 mL extract was mixed with 9 mL of pH 1.0 and pH 4.5 buffers in separate conditions. In pH 1.0 and 4.5 buffers, absorbance was measured at 520 and 700 nm, expressing the result (cyanidin 3-glucoside) using Equation (6).

(6) $$A={\left(A_{510}-A_{700}\right)}_{\mathrm{pH}\;1.0}-{\left(A_{510}-A_{700}\right)}_{\mathrm{pH}\;4}$$

(7) $$\mathrm{Total}\;\mathrm{monomeric}\;\mathrm{anthocyanin}\;(\mathrm{mg}/\mathrm{mL})\;=\;\frac{A\;\times \;\mathrm{MW}\;\times \;\mathrm{DF}}{\varepsilon \;\times \;L}$$

where *A* = absorbance, *ε* = cyd-3-glucoside molar absorbance (26,900), MW = anthocyanin molecular weight (449.2), DF = dilution factor, and *L* = cell path length (1 cm). Final results are expressed as equivalent per gram dry matter (mg C_3_gE/g DM).

#### 2.5.3. Radical-Scavenging Activity (RSA)

In triplicate, the RSA assay was carried out according to Fawole and Opara [1]. Briefly, in test tubes, an aqueous methanolic extract of PJP (0.015 mL) was diluted with methanol (0.735 mL) and methanolic DPPH solution (0.75 mL, 0.1 mM) was immediately added. The mixtures were incubated in the dark and at room temperature for 30 min. The absorbance was measured at 517 nm using a UV-vis spectrophotometer (Helios Omega, Thermo Scientific, Waltham, MA, USA) and compared with the standard curve (Trolox equivalent, 0–2.0 mM). The free-radical activity of PJP was expressed as Trolox equivalent (mM) per gram dry matter (mM TE/g DM).

#### 2.5.4. Ferric-Ion Reducing Antioxidant Power (FRAP)

The antioxidant power of PJP was measured using the calorimetric method, according to [1,37]. The FRAP working solution was freshly prepared in mixtures of 300 mM acetate buffer (50 mL), 10 mM 2,4,6-tripyridyl-s-triazine (TPTZ; 5 mL) and 20 mM ferric chloride (5 mL) at 37 °C. Diluted aqueous methanolic PJP extracts (0.15 mL) were added to 2.85 mL of the FRAP working solution in triplicates, followed by incubating the mixture in the dark for 30 min. Measurement of the absorbance at 593 nm was carried out to monitor the reduction of the Fe^3+^-TPTZ complex to a coloured Fe^2+^-TPTZ complex at low pH by PJP extracts. Trolox (0–10 mM) was used for the calibration curve, and the results were expressed as Trolox (mM) equivalents per gram dry matter (mM TE/g DM).

### 2.6. Statistical Analysis

Data were analysed using STATISTICA (Statistica 13.0, StatSoft Inc., Tulsa, OK, USA) and presented as means ± standard error. All analyses were done in triplicates. Data were subjected to analysis of variance (ANOVA), and means were separated according to Fisher’s LSD test at a level of significance of 95%. The graphical presentations were processed by using GraphPad Prism software 4.03 (GraphPad Software, Inc., San Diego, CA, USA). Principal component analysis (PCA) was carried out using XLSTAT software version 2012.04.1 (Addinsoft, Bordeaux, France).

## 3. Results and Discussion

### 3.1. Quality Attributes of Pomegranate Juice

Table 1 shows the quality attributes of the pomegranate juice used for freeze-drying. It was observed that the pomegranate juice had strong acidity (pH value 3.10), which implied that it is less susceptible to microbial growth. The high level of TSSs (15.3 °Brix) is typical of the pomegranate cv. Wonderful as it is considered as sweet-sour cultivar [38]. Predominant sugars in pomegranate juice are glucose, fructose and sucrose [39] and are reported to contribute to powder stickiness during drying [21,40]. The phenolic contents (TPC and TAC) have additive effects on the pharmacological properties of pomegranates and gives the characteristic red colouration to the juice [39,41]. Values of TPC and TAC correspond with that of [38]. The contents of antioxidants serve as indicators for nutrient retention during the freeze-drying process. Depending on the cultivar, the colour of pomegranate juice could range from very deep to slightly red. Based on the moderately low values for the colour parameters (L*, a*, C* and *h*°), the investigated pomegranate juice could be considered as slightly red in colour. Properties of colour are quality indicators that are necessary to reflect sensory attractiveness; thus, the production of phenolic-rich coloured PJP was of high priority in this study.

### 3.2. Yield, Moisture Content and Water Activity

The effect of carrier agents on the yield of freeze-dried PJP is presented in Table 2. The most amount of powder was obtained with maltodextrin (46.6%), while waxy starch yielded the least (35.4%). This disagrees with Yousefi et al. [28], who reported that gum arabic had the highest yield in their study of the effect of carrier type and spray drying of pomegranate juice. This could be due to differences in the drying methods of the production of PJP. Additionally, the differences in the yield of PJP could be due to the configuration of the carrier agents. The least yield found in waxy starch was also noted by Yousefi et al. [28] due to its crystalline nature.

The moisture content (MC) and water activity (*a*_w_) of freeze-dried PJP were significantly (*p* < 0.05) different among carriers (Table 2). Gum-arabic-added PJP had the highest moisture (1.8%) after drying, while waxy-starch-added PJP had the least value of moisture (0.2%). Similarly, the highest values of water activity were observed in the powder produced with gum arabic (0.49), followed by maltodextrin-added PJP, which had 0.31, while waxy starch-added PJP had the least water activity (0.20).

In dried food materials, moisture content is one of the factors related to drying efficiency [31]. From this study, the powder produced with waxy starch had low moisture (*p* < 0.05) and water activity and was strongly and positively correlated (*r* = 0.947). This was expected as the decrease in moisture content could directly be linked to reduced water activity and, therefore, the powder produced with waxy starch could perform better in storage stability than powders obtained with other drying agents. Decreased water activity prevents the growth of most bacteria, yeasts, and moulds, which are not capable of growing below water activity values of 0.87, 0.88, and 0.80, respectively [42]. Furthermore, Laroche et al. [43] noted that water activity values ranging between 0.20–0.50 prevented microbial infestation in food powders. Daza et al. [44] also reported that freeze-dried samples with values of water activity lower than 0.3 were less susceptible to microbial attack. Results from this study also support the findings by Mosquera et al. [45], who reported that the lower the critical water activity in freeze-dried strawberry powder, the better the stability during storage. Lower moisture prevents the agglomeration of particles which hinders the caking of powder, thereby reducing the retention of active components and other powder properties such as flowability and dispersion [46]. Lower moisture content in dried fruit is related to its low water activity [47]. The high moisture content and water activity observed in powder produced with gum arabic may be explained due to the difficulty for water to diffuse through the carrier agent, where crusts are formed around the surface particle [48].

### 3.3. Colour Attributes

Fresh pomegranate juice has a favourable red colour due to the rich content of anthocyanins [1]. Lightness (L*) of freeze-dried PJP was significantly (*p* < 0.05) different among carrier agents (Table 2). Waxy starch appeared lighter (78.7) than maltodextrin and gum arabic, with lightness values of 69.0 and 64.6, respectively. According to Comunian et al. [49], the increased lightness in powder obtained from waxy starch was as a result of the dilution effect, which was the pure white colour of the carrier, and this could be responsible for the lighter colour of the PJP. In contrast, the darker colour of gum arabic could be responsible for the darker red colour of the powder. The effect of carrier type on the quality properties of powdered and reconstituted pomegranate juice (cv. Malas) was previously assessed by Yousefi et al. [28] and an increase in the values of L* with the use of waxy starch and maltodextrin was observed in comparison to gum arabic.

The characteristic red colouration of pomegranate powder measured as a* was also significantly (*p* < 0.05) different among carrier agents (Table 2). Juice powder produced with maltodextrin had the highest redness (29.3), while gum arabic had the least value (16.3). Furthermore, a significant (*p* < 0.05) difference was observed between carrier agents for chroma (C*) of freeze-dried pomegranate powder (Table 2). PJP produced with maltodextrin had the highest C*, followed by those produced by waxy starch and gum arabic. Hue angle (*h*^o^) of PJP was significantly (*p* < 0.05) different among carrier agents. PJP produced with maltodextrin had the least colour purity (*h*°; 0.6), closer to 0°, which suggests a higher degree of redness compared to PJP produced with gum Arabic, with highest hue angle (11.9). This suggests that changes observed in the red colour of PJP depend on the type of carrier agent [14,50]. The powder produced with waxy starch had the highest TCD (56.5), followed by maltodextrin (49.2) while powders produced with waxy starch showed the lowest TCD (42.2). Overall, the changes in colour attributes could be attributed to the addition of carrier and alteration in polyphenols during drying [51].

### 3.4. Total Soluble Solids (TSSs) and Titratable Acidity (TA)

Table 3 shows the effect of carrier agents on the total soluble solids (TSSs), titratable acidity (TA) and pH of PJP.

PJP produced with maltodextrin and gum arabic had higher TSSs (10.3 and 10.4 °Brix, respectively) than those produced with waxy starch (8.6 °Brix; Table 3). Increased soluble solids composition observed for PJP produced with maltodextrin and gum arabic were approximately 17% higher than waxy starch. Similarly, there was a significant (*p* < 0.05) difference among carrier agents in the titratable acidity (TA) of PJP (Table 3). PJP produced with maltodextrin had the highest TA (0.24%), while powder produced with waxy starch had the least (0.18%). Rahman and Lamb [52] stated that soluble solids, organic acids, amino acids, soluble pectin and mineral salts, among several others, are the major chemical constituents found in fruit. Higher values of total soluble solids and titratable acidity observed in the powders produced with maltodextrin and gum arabic can be attributed to the crystalline nature of carrier agents. Carrier agents or additives are different in molecular weight compounds and crystalline configuration [28,53,54,55].

### 3.5. Technofunctional Properties

#### 3.5.1. Solubility

The solubility index is an important feature to characterise the wettability and dispersibility of powders in solutions. Solubility differed significantly (*p* < 0.05) among the juice powders (Table 4).

Powders produced with maltodextrin and gum arabic showed similar results with higher solubility (96.5 and 96.1%, respectively), while waxy starch had the least solubility (35.4%). Higher values observed in the powders produced with maltodextrin and gum arabic could be related to the crystalline nature of the powder. Cano-Chauca et al. [24], in their study on spray drying of mango juice powder, also recorded a higher value of up to 95% for maltodextrin- and gum-arabic-produced powders, which is similar to the values generated in this study. Similarly, the solubility values of pineapple and cashew juice powders were also higher, with average values of 81.56 and 95.1%, respectively [56,57]. The low solubility value observed in waxy starch, reported in this study, was also supported by Mishra and Rai [58], who reported less solubility in the powder produced with waxy starch.

#### 3.5.2. Hygroscopicity

Table 4 shows significant (*p* < 0.05) differences among carrier agents in the hygroscopic nature of freeze-dried PJP. The powder produced with gum arabic had the highest hygroscopicity (12.6%), followed by maltodextrin (10.2%), while samples produced with waxy starch showed the lowest hygroscopicity (4.7%). Differences in hygroscopic values could be due to the nature of the powders and the rate at which the powders produced hold molecules of water from the surrounding air. The result from this study was similar to the study by Tonon et al. [59], who reported that gum arabic showed the highest percentage of hygroscopicity in comparison with maltodextrin 10DE, maltodextrin 20DE and tapioca starch in acai powder. The authors noted that hygroscopicity of powder could be used to explain the mechanisms of water adsorption in powder materials as being attributed to the number of hydrophilic groups appearing in the structure of each carrier.

Furthermore, a higher number of hydrophilic groups are present in maltodextrin and gum arabic, which relates to the easy absorption of moisture from the atmosphere [60]. The authors also explained that the dynamics of moisture adsorption by carbohydrate material is duly associated with the links between the hydrogen available in the molecules of water and the hydroxyl groups present in the amorphous region of the substrate and the crystalline region. Similarly, a high hygroscopic nature of powder as a result of water absorbed from the surrounding air could also be used to explain the high moisture content of the powder [14]. However, the moisture–hygroscopicity relationship cannot be made general for all powder samples. For instance, a study by Ahmed et al. [61] noted that the hygroscopic nature of spray-dried sweet potato was highly influenced by drying agents and could not be directly related to varying moisture content.

#### 3.5.3. Bulk Density

The results of the bulk density of freeze-dried PJP produced with different carrier agents are also shown in Table 4. Powders produced with maltodextrin exhibited the highest value of bulk density (0.77 g cm^−3^), followed by gum arabic (0.74 g cm^−3^), while waxy starch had the least bulk density (0.64 g cm^−3^). Bulk density is the “addition of the mass of solid particles and moisture per total volume occupied by the particles, surface moisture and all pores, closed or open, in the surrounding atmosphere and is generally used to characterise the final product obtained by milling or drying” [28,62]. Furthermore, Chegini and Ghobadian [63] reported that powder with higher moisture content is usually associated with higher bulking weight because of the minute volume of water attracted from the atmosphere, which is considerably denser than the dry solid material. This report is in line with the results obtained in this study. PJP produced with maltodextrin and gum arabic showed higher moisture content and higher bulk density. This also supported the findings by Ferrari et al. [35], who related a higher moisture content with bulk density in powders produced with gum arabic as well as the mixture of both maltodextrin and gum arabic.

#### 3.5.4. Water- and Oil-Holding Capacity

There was a significant (*p* < 0.05) difference among carrier agents in the water-holding capacity (WHC) of freeze-dried PJP (Table 4). Results showed that waxy starch had the highest WHC (1.84 g/g), while gum arabic had the least (0.25 g/g). Furthermore, it was observed that waxy starch held water for up to 63.6% and 86.4% more than maltodextrin and gum arabic, respectively. The higher performance of waxy starch to hold more water than other carrier agents could be attributed to the particle structure of the carrier agents. An increase in the particle density of the powder is associated with a reduction in water-holding capacity [64]. Hong and Zhang [65] also reported similar results in their study on the effect of ultra-fine pulverisation on the particle structure of soybean dietary fibre.

There was a significant (*p* < 0.05) difference among carrier agents in the oil-holding capacity (OHC) of freeze-dried PJP (Table 4). Gum arabic had the highest values for oil-holding capacity (1.96 mL/g), followed by maltodextrin with 1.64 mL/g, while waxy starch had the least (1.45 mL/g). This indicates that the higher the WHC of PJP, the lower the OHC for the studied carrier agents. The nature of the carrier agents could also be related to the OHC of PJP. A study by Chau et al. [66] noted that particle size and processing technique, such as the addition of additives or carriers, could be traceable to the effective increase in the oil-holding capacity of powder.

#### 3.5.5. Particle Size Distribution

**Figure 2:**
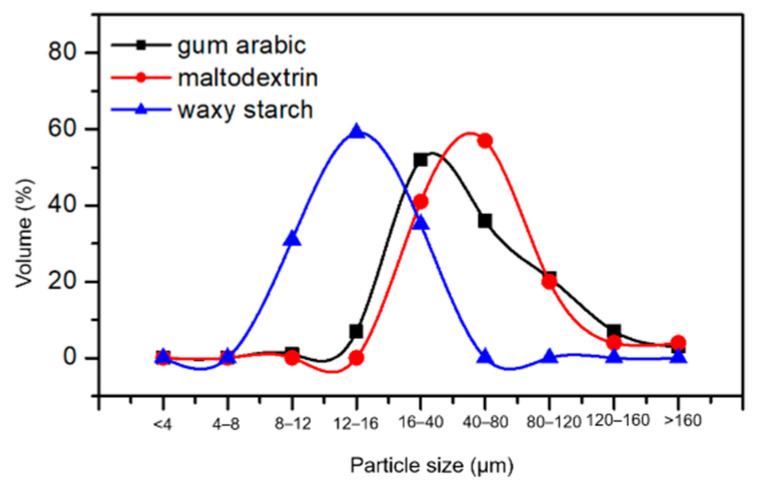
Particle size distribution of freeze-dried pomegranate powders produced with different carriers.

Figure 2 shows the particle size distribution of freeze-dried pomegranate powders obtained using maltodextrin, gum arabic and waxy starch as carrier agents. A normal distribution curve was observed for all the carriers. The particles produced with waxy starch showed the highest volume (59.8%) and the lowest particle diameter within the range of 8 to 40 µm. The powder produced with maltodextrin and gum arabic presented volumes of 59.3% and 52.5%, respectively, while their particle diameters ranged between 12 and 120 µm.

Particles obtained from maltodextrin and gum arabic exhibited larger size ranges that are normal in the case of powder analysis since a higher proportion of smaller particles occupies or fills up the spaces in between the larger ones. The formation of larger particles is not only attributed to agglomeration but also the molecular size of the carriers [59]. The process of agglomeration breaks down the powders’ exposure to oxygen, and, thereby, anthocyanin pigments are protected. The higher retention of anthocyanin in powders formed with maltodextrin and gum arabic could be explained by the characteristic feature of powder agglomeration [31]. The mean diameter of pomegranate powders obtained with maltodextrin and gum arabic were different to that of other fruit powders such as blackberry (13.0–34.2 µm), and raspberry (14.6–18.3 µm) [67,68]. This could be due to the low operating temperature of the freeze-dryer, which makes the initial phase of agglomeration easier as a result of irreversibly bound particles during drying, resulting in larger particle sizes [67]. Additionally, according to Kurozawa et al. [69], the solubility and flowability of spray-dried powder reduced with a decrease in the size of the particle. This is similar to the observed solubility result presented in this study (Table 4). A direct particle size-solubility relationship was observed in the present study.

#### 3.5.6. Microstructure

**Figure 3:**
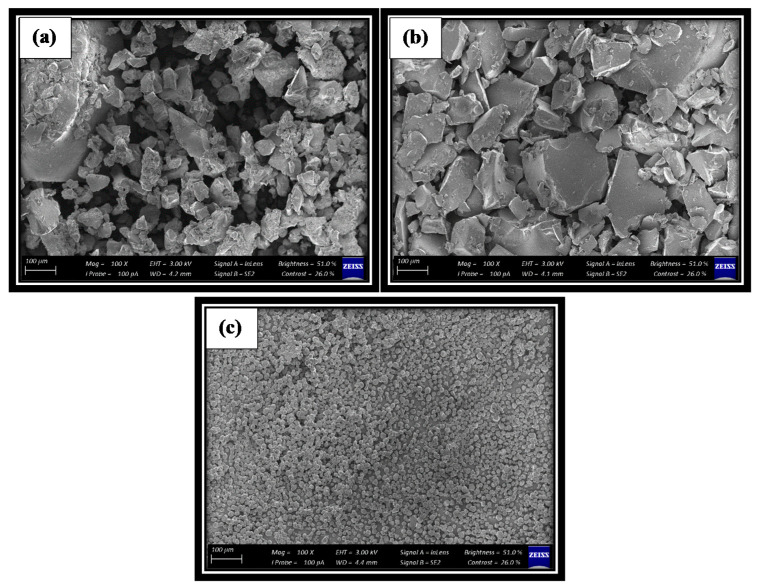
Scanning electron microscopy (SEM) microphotographs of freeze-dried pomegranate powder prepared with (**a**) maltodextrin, (**b**) gum arabic and (**c**) waxy starch.

Figure 3 presents the scanning electron microscopy (SEM) microstructure of the powders produced with different carrier agents. The powders produced had particles of different sizes for the carrier agents. Powder production using maltodextrin and gum arabic resulted in the smaller particles adhering strongly to the surface of the larger ones (agglomeration), which is in agreement with the results obtained for particle size distribution. There were similarities in the powders produced with maltodextrin and gum Arabic, both showing predominantly angular shapes, while particles prepared with waxy starch have a higher degree of uniformity with a spherical shape, as shown in Figure 3c. This result also supported the findings by Leonel [70], who evaluated tapioca starch morphology and observed a rounded shape and smooth surface. Loksuwan [71] also reported a similar structure when investigating the morphology of tapioca starch during β-carotene encapsulation.

### 3.6. Total Phenolic and Anthocyanin Contents

**Figure 4:**
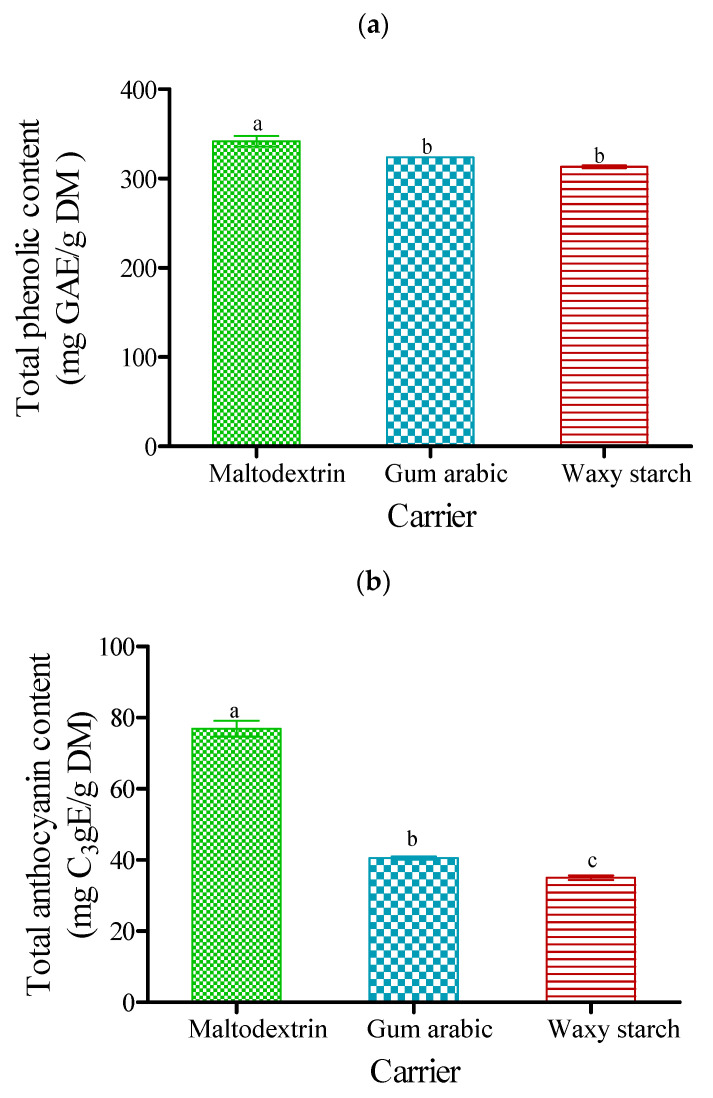
Total phenolic content (**a**) and total anthocyanin content (**b**) of freeze-dried pomegranate powder using different carriers. Bars with diﬀerent letters are significantly different (*p* < 0.05) according to Fisher’s LSD. Vertical bars indicate the standard error of the mean.

Graphical representation of total phenolic content and total anthocyanin content observed is shown in Figure 4a,b.

Results indicated that total phenolic content (TPC) of freeze-dried PJP was significantly (*p* < 0.05) different among carrier agents (Figure 4a). PJP produced with maltodextrin had the highest TPC (341.8 mg GAE/g DM); however, PJP produced with gum arabic resulted in a lower total phenolic content (323.8 mg GAE/g DM) that was not statistically different from the powder produced with waxy starch, which had the least TPC (313.3 mg GAE/g DM). Furthermore, total phenolic content was approximately 8.3% more in the powder produced with maltodextrin than waxy starch.

As observed in this study, the carrier agents related differently to the production of PJP, which could be a result of their structural complexities due to their soluble or insoluble nature. For instance, the nature of powder produced with maltodextrin and gum arabic appeared coarse with larger particle sizes, where the specific surface area could be lower than powder produced with waxy starch, with a finer appearance. Results showed that the interactions might interfere with polyphenol extraction and determination in the powder samples. Du et al. [14] noted the different interactions in three carbohydrate carriers (maltodextrin, gum arabic and starch sodium octenyl succinate) in the production of persimmon pulp powders. The authors noted that carriers showing the least polyphenol retention had the smallest particle size, with more surfaces exposed to oxygen, thus resulting in lower polyphenol retention. However, Tonon et al. [59] reported that gum arabic showed greater potential compared to maltodextrin and tapioca starch with regard to polyphenol retention in spray-dried acai pulp powders. This markedly distinct character may be due to the different samples, structure of carriers, and drying condition used.

Similarly, for the total anthocyanin content (TAC), there was a significant (*p* < 0.05) difference among carrier agents (Figure 4b). The powder produced with maltodextrin had the highest TAC (76.91 mg C_3_gE/g DM), whereas waxy starch had the lowest TAC (35.01 mg C_3_gE/g DM). Furthermore, an approximate 54% higher in total anthocyanin content was observed for maltodextrin compared to waxy starch. Yousefi et al. [28] investigated the use of maltodextrin, gum arabic, and waxy starch as carrier agents in the spray drying of pomegranate juice. The authors found that maltodextrin was more effective than gum arabic and waxy starch with regard to the preservation of anthocyanins, which supports the result of this study. Similarly, Tonon et al. [72] reported the lowest anthocyanin retention in the powder produced with starch.

### 3.7. DPPH Radical Scavenging Activity and FRAP Activity

**Figure 5:**
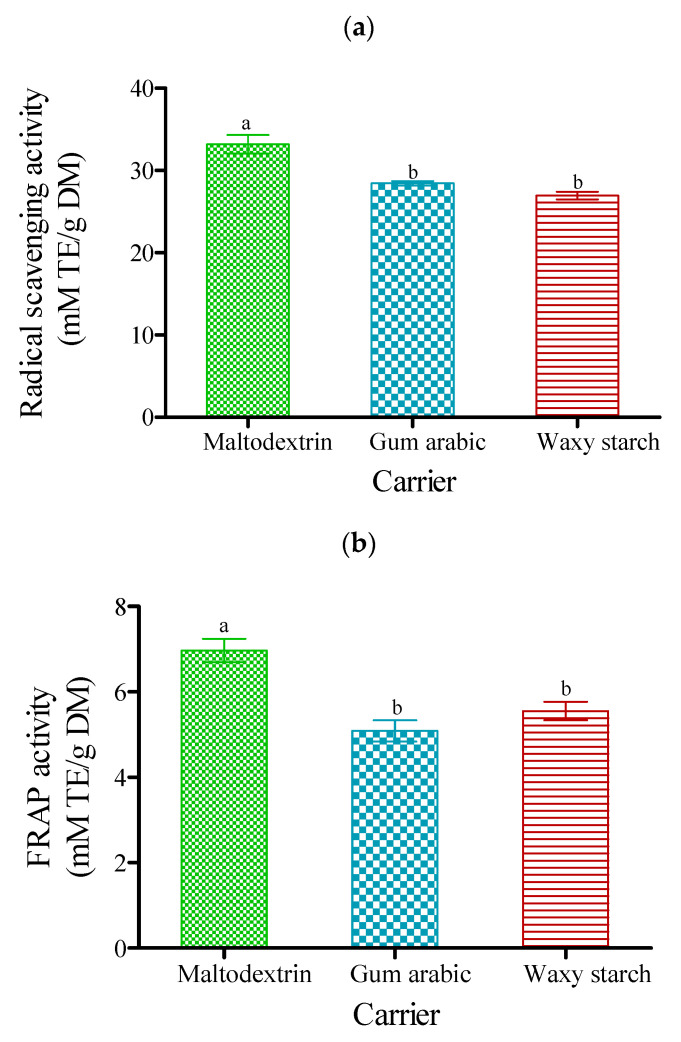
Antioxidant capacity (**a**) radical scavenging activity (RSA) and (**b**) ferric-reducing antioxidant power (FRAP) activity of freeze-dried pomegranate powder using different carriers. Bars with diﬀerent letters are significantly different (*p* < 0.05) according to Fisher’s LSD. Vertical bars indicate the standard error of the mean.

The radical scavenging activity (RSA) of PJP was significantly (*p* < 0.05) different among carrier agents (Figure 5a). The powder produced with maltodextrin had higher antioxidant capacity RSA (33.19 mM TE/g DM), compared to gum arabic (28.45 mM TE/g DM) and waxy starch (26.96 mM TE/g DM). The lower value in powder produced with waxy starch could be traceable to the insoluble nature of the carrier.

Similarly, the ferric-reducing power (FRAP) of PJP was significantly (*p* < 0.05) different among carrier agents (Figure 5b). PJP produced with maltodextrin had the highest FRAP (6.97 mM TE/g DM), while powder produced with gum arabic had the least (5.09 mM TE/g DM). The higher value of the powder produced with maltodextrin could be traceable to the high soluble nature of the carrier agent. Tonon et al. [72] reported that powders produced with maltodextrin showed higher antioxidant capacity in the spray-dried acai powder due to its high soluble nature, which supports the results of this study. Lim et al. [73] also noted that powder produced with maltodextrin increased the antioxidant capacity of spray-dried blueberry by-products. Furthermore, maltodextrin as a drying agent significantly increased the antioxidant activity of spray-dried amla juice powder [74].

### 3.8. Principal Component Analysis

**Figure 6:**
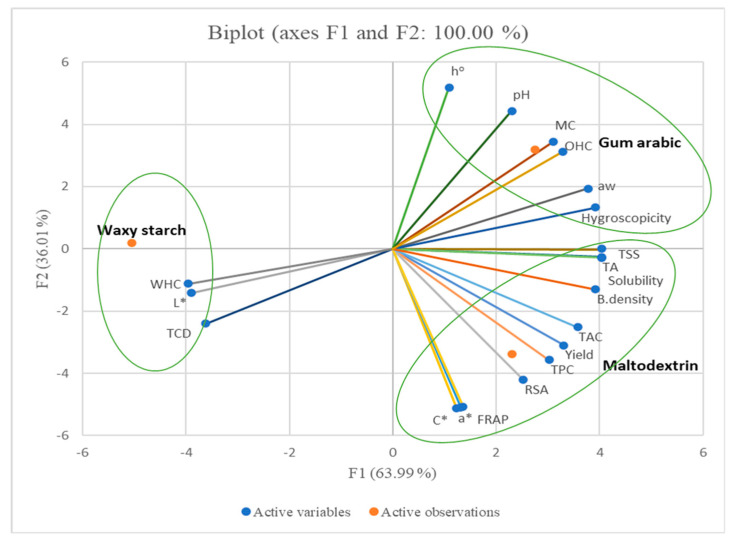
Principal component analysis of the first two factors (F1 and F2) based on physicochemical properties, phenolic contents, antioxidant capacity and technofunctional properties of pomegranate powder cv. Wonderful obtained from maltodextrin, gum arabic and waxy starch. MC, moisture content; L*, lightness; a*, redness; C*, chroma; *h*°, hue angle; TCD, total colour difference; RSA, radical scavenging activity; FRAP, ferric reducing antioxidant power; TPC, total phenolic content; TAC, total anthocyanin content; TSSs, total soluble solids; TA, titratable acidity; *a*_w_, water activity; WHC, water holding capacity; OHC, oil holding capacity.

The results show the average of phenolic contents, antioxidant capacity, technofunctional properties and colour coordinates of pomegranate of freeze-dried powder. The two principal components (F1 and F2) explain 100.0% of the total data variance (Figure 6).

As observed, F1 explained 63.99% of the total variance, while F2 explained only 36.01% of the total variability, which showed that the disparity among freeze-dried pomegranate powder was described by F1 (Figure 6). The observations indicated that the powders produced with maltodextrin and gum arabic had higher positive scores along the F1 plane and could be associated with moisture content (MC), water activity (*a*_w_), total soluble solids (TSSs), titratable acidity (TA), pH, yield, total phenolic content (TPC), radical scavenging activity (RSA), total anthocyanin content (TAC), solubility, hygroscopicity, bulk density and oil-holding capacity (OHC) (Table 5).

In addition, higher negative scores along F1 (Table 5; Figure 6) correspond to TCD, water-holding capacity and the lightness of the powder produced with waxy starch. Along F1 (Figure 6), lower positive scores correspond to redness (a*), chroma (C*), hue (*h*°) and ferric-reducing antioxidant power (FRAP) of freeze-dried powder produced with maltodextrin and gum arabic. Likewise, high positive scores along F2 (Table 5) is associated with hue (*h*°), moisture content (MC), pH and oil-holding capacity (OHC) of the freeze-dried powder produced with gum arabic (Figure 6). Additionally, along the F2, high negative scores (as shown in Figure 6 and Table 5) for maltodextrin could characterise the powder for having high yield, redness (a*), chroma (C*), total phenolic content (TPC), radical scavenging activity (RSA) and ferric-reducing antioxidant power (FRAP). However, lower positive scores along F2 were from freeze-dried powder from gum arabic (associated with water activity (*a*_w_), total soluble solids (TSSs) and hygroscopicity). The lower negative scores (Figure 6) along F2 (Table 5) were from maltodextrin and waxy starch (associated with lightness (L*), TCD, titratable acidity (TA), solubility, total anthocyanin content (TAC), bulk density and water-holding capacity (WHC). The results from the PCA showed that powders produced with carrier agents (maltodextrin, gum arabic and waxy starch) resulted in significantly different properties.

## 4. Conclusions

The use of three carrier agents (maltodextrin, gum arabic and waxy starch) in the production of freeze-dried PJP was investigated. The results indicated that maltodextrin was more effective in enhancing the yield as well as the physicochemical properties of the PJP, such as colour, TSSs and TA. Similarly, maltodextrin and gum arabic performed better as carriers agents in enhancing the solubility of freeze-dried PJP compared to waxy starch. Maltodextrin was better in the preservation of phenolic content and antioxidant capacity of PJP. Therefore, it could be inferred that maltodextrin resulted in the best carrier agent that retained biochemical activities and maintained the technofunctional properties in the production of freeze-dried pomegranate powder. This study has shown that maltodextrin is the most suitable carrier agent for the formulation or fortification of pomegranate-based food products for baking, candies and ice-cream. This study reports the results of powder produced in a laboratory-scale freeze-dryer. However, a scale-up can be investigated in order to produce, on an industrial scale, powders with similar characteristics. Moreover, further research is required to investigate the storability and optimisation of PJP.

## Figures and Tables

**Table 1:** Quality attributes of pomegranate juice (cv Wonderful) processed into powders.

Quality Attributes	Values (Means ± SE)
TSS (°Brix)	15.3 ± 0.07
TA (% citric acid)	1.35 ± 0.01
pH	3.10 ± 0.01
TPC (mg GAE/100 mL)	22.6 ± 2.82
TAC (mg C_3_gE/100 mL)	7.4 ± 1.95
L*	23.0 ± 2.21
a*	15.4 ± 1.37
C*	18.6 ± 1.44
*h*°	34.5 ± 0.94

L*, lightness; a* redness; C* chroma; *h*°, hue angle; TSSs, total soluble solids; TA, titratable acidity; TPC, total phenolic content; TAC, total anthocyanin content. SE: standard error.

**Table 2:** Yield, moisture content, water activity and colour attributes of freeze-dried pomegranate juice powder (PJP).

Carrier	Yield %	MC %	*a*_w_	L*	a*	C*	*h*°	TCD
Maltodextrin	46.6 ± 0.04 ^a^	0.7 ± 0.02 ^b^	0.31 ± 0.00 ^b^	69.0 ± 1.42 ^b^	29.3 ± 0.49 ^a^	29.3 ± 0.48 ^a^	0.6 ± 0.19 ^c^	49.2 ± 1.39 ^b^
Gum arabic	40.6 ± 0.12 ^b^	1.8 ± 0.02 ^a^	0.49 ± 0.01 ^a^	64.6 ± 0.39 ^c^	16.3 ± 0.24 ^c^	16.7 ± 0.23 ^c^	11.9 ± 0.37 ^a^	42.2 ± 0.40 ^c^
Waxy starch	35.4 ± 0.30 ^c^	0.2 ± 0.02 ^c^	0.05 ± 0.00 ^c^	78.7 ± 1.20 ^a^	18.6 ± 0.29 ^b^	18.6 ± 0.29 ^b^	4.0 ± 0.31 ^b^	56.5 ± 1.19 ^a^

MC, moisture content; L*, lightness; a* redness; C* chroma; *h*°, hue angle; *a*_w_, water activity; TCD, total colour difference. Presented as means ± SE in each column, data followed by different letters are significantly different (*p* < 0.05) according to Fisher’s LSD.

**Table 3:** Physicochemical attributes of freeze-dried PJP.

Carrier	TSSs (°Brix)	TA (% Citric Acid)
Maltodextrin	10.3 ± 0.17 ^a^	0.24 ± 0.04 ^a^
Gum arabic	10.4 ± 0.21 ^a^	0.24 ± 0.02 ^a^
Waxy starch	8.6 ± 0.20 ^b^	0.18 ± 0.01 ^b^

TSSs, total soluble solids; TA, titratable acidity. Presented as means ± SE in each column, data followed by different letters are significantly different (*p* < 0.05), according to Fisher’s LSD.

**Table 4:** Technofunctional properties of freeze-dried PJP.

Carrier	Solubility %	Hygroscopicity %	Bulk Density (g cm^−3^)	WHC (g/g)	OHC (g/g)
Maltodextrin	96.5 ± 0.09 ^a^	10.2 ± 0.04 ^b^	0.77 ± 0.01 ^a^	0.67 ± 0.03 ^b^	1.64 ± 0.01 ^b^
Gum arabic	96.1 ± 0.46 ^a^	12.6 ± 0.01 ^a^	0.74 ± 0.02 ^a^	0.25 ± 0.01 ^c^	1.96 ± 0.01 ^a^
Waxy starch	35.4 ± 0.09 ^b^	4.7 ± 0.07 ^c^	0.64 ± 0.02 ^b^	1.84 ± 0.01 ^a^	1.45 ± 0.01 ^c^

Presented as means ± SE in each column, data followed by different letters are significantly different (*p* < 0.05), according to Fisher’s LSD.

**Table 5:** Factor loadings, eigenvalue, cumulative variance (%) and score for the first two principal (F1–F2) components based on different carrier agents.

Loadings	F1	F2
L*	−0.965	−0.263
a*	0.306	−0.952
C*	0.324	−0.946
*h*°	0.270	0.963
TCD	−0.895	−0.446
MC	0.769	0.640
*a*_w_	0.933	0.359
TSS	1.000	0.000
TA	0.999	−0.050
pH	0.570	0.822
Yield	0.818	−0.576
TPC	0.748	−0.664
RSA	0.624	−0.781
FRAP	0.336	−0.942
TAC	0.884	−0.468
Solubility	0.999	−0.054
Hygroscopicity	0.969	0.247
Bulk density	0.970	−0.243
WHC	-0.978	−0.208
OHC	0.815	0.580
Scores		
Maltodextrin	2.309	−3.377
Gum arabic	2.744	3.188
Waxy starch	−5.053	0.189

MC, moisture content; L*, lightness; a*, redness; C*, chroma; *h*°, hue angle; TCD, total colour difference; RSA, radical scavenging activity; FRAP, ferric reducing antioxidant power; TPC, total phenolic content; TAC, total anthocyanin content; TSSs, total soluble solids; TA, titratable acidity; *a*_w_, water activity; WHC, water-holding capacity; OHC, oil-holding capacity.

## Abbreviations

PJP	pomegranate juice powder
MC	moisture content
L*	lightness
a*	redness
C*	chroma
h°	hue angle
TCD	total colour difference
RSA	radical scavenging activity
FRAP	ferric reducing antioxidant power
TPC	total phenolic content
TAC	total anthocyanin content
TSS	total soluble solids
TA	titratable acidity
a_w_	water activity
WHC	water holding capacity
OHC	oil holding capacity
SEM	scanning electron microscopy
w.b.	wet basis
RH	relative humidity
